# Performance Analysis of Conventional Machine Learning Algorithms for Diabetic Sensorimotor Polyneuropathy Severity Classification

**DOI:** 10.3390/diagnostics11050801

**Published:** 2021-04-28

**Authors:** Fahmida Haque, Mamun Bin Ibne Reaz, Muhammad Enamul Hoque Chowdhury, Geetika Srivastava, Sawal Hamid Md Ali, Ahmad Ashrif A. Bakar, Mohammad Arif Sobhan Bhuiyan

**Affiliations:** 1 Department of Electrical, Electronic and System Engineering, Universiti Kebangsaan Malaysia, Bangi 43600, Malaysia; fahmida32@yahoo.com (F.H.); mamun@ukm.edu.my (M.B.I.R.); sawal@ukm.edu.my (S.H.M.A.); ashrif@ukm.edu.my (A.A.A.B.); 2 Department of Electrical Engineering, Qatar University, Doha 2713, Qatar; mchowdhury@qu.edu.qa; 3 Department of Physics and Electronics, Dr. Ram Manohar Lohia Avadh University, Ayodhya 224001, India; geetika_gkp@rediffmail.com; 4 Department Electrical and Electronic Engineering, Xiamen University Malaysia, Bandar Sunsuria, Sepang 43900, Malaysia

**Keywords:** DSPN, ML, severity classification, machine learning, diabetic neuropathy, MNSI

## Abstract

Background: Diabetic peripheral neuropathy (DSPN), a major form of diabetic neuropathy, is a complication that arises in long-term diabetic patients. Even though the application of machine learning (ML) in disease diagnosis is a very common and well-established field of research, its application in diabetic peripheral neuropathy (DSPN) diagnosis using composite scoring techniques like Michigan Neuropathy Screening Instrumentation (MNSI), is very limited in the existing literature. Method: In this study, the MNSI data were collected from the Epidemiology of Diabetes Interventions and Complications (EDIC) clinical trials. Two different datasets with different MNSI variable combinations based on the results from the eXtreme Gradient Boosting feature ranking technique were used to analyze the performance of eight different conventional ML algorithms. Results: The random forest (RF) classifier outperformed other ML models for both datasets. However, all ML models showed almost perfect reliability based on Kappa statistics and a high correlation between the predicted output and actual class of the EDIC patients when all six MNSI variables were considered as inputs. Conclusions: This study suggests that the RF algorithm-based classifier using all MNSI variables can help to predict the DSPN severity which will help to enhance the medical facilities for diabetic patients.

## 1. Introduction

Diabetes mellitus (DM), one of the fastest rising health concerns of the 21st century. The number of patients affected with DM worldwide has increased from 151 million in 2000 to 463 million in 2019; over just 20 years [1]. International Diabetic Federation (IFD) estimated that globally by 2045, approximately 700 million people will be affected by diabetes [1]. DM is a common yet costly metabolic disease, which leads to serious damage to different organs of the body with the long-term uncontrolled blood glucose level [2,3,4,5]. Among all the complications that arise due to DM, diabetic sensorimotor polyneuropathy (DSPN) a very common form of neuropathy caused by diabetes. It affects limb nerves, especially in the lower limb. Globally, 40 to 60 million people with diabetes are suffering from lower limb complications due to DSPN [1]. Long-term DSPN leads to ulceration and amputations, significantly increasing the chance of early death and reducing the quality of life. Globally, every 30 s, one lower limb amputation is happening due to DSPN [6]. Henceforth, early identification of DSPN to provide proper treatment to prevent the life-threatening condition is inevitable. According to the study [7], less than one-third of health physicians would be able to identify the signs of DSPN, resulting in misleading diagnoses, contributing to high rates of morbidity and mortality. Although a variety of screening and diagnosing methods are accessible for DSPN, most of them require expensive equipment and specialized personnel to analyze the results from these tests; some of the methods are invasive and painful; some are not reproducible, and some have contradictory outcomes due to lack of standardization in diagnosis measures. Moreover, among the health professionals, there is a gap in understanding regarding the thorough diagnosis, controlling, and medication process for DSPN [8]. Therefore, for early identification and satisfaction of the DSPN severity, a simple, cost-effective, reproducible, accurate diagnosis method is necessary, which can be globally applicable to solve the lack of understanding among the health professionals regarding DSPN.

According to the 19th annual Diabetic Neuropathy Study Group of the European Association for the Study of Diabetes (NEURODIAB) and the 8th International Symposium on Diabetic Neuropathy in Toronto, Canada, 2009, DSPN has been categorized as ‘possible DSPN’ when the presence of either symptoms or signs of neuropathy establishes; ‘probable DSPN’ is diagnosed only in the presence of symptoms and signs of neuropathy; ‘confirmed DSPN’ was considered when symptoms or signs of neuropathy were present with abnormal nerve conduction studies (NCS); and, finally, ‘subclinical DSPN’ was diagnosed with the presence of abnormal NCS without symptoms or signs. NCS is considered as the benchmark for the identification and stratification of DSPN [9]. Based on NCS, signs, and symptoms severity grading are performed for DSPN [10]. However, NCS is an expensive procedure, requires expertise and specialized equipment that might not be available in all of the regular healthcare centers. Therefore, patients are screened for signs and symptoms of DSPN and based on the results patients are classified as different levels of DSPN severity and sent for NCS if the patients exhibit severe symptoms and signs of neuropathy.

According to American Diabetic Association (ADA), for accurate identification of DSPN, the diagnosis should be combined with clinical history, a physical examination for signs, and electrophysiological screening such as nerve conduction studies (NCS) [11,12,13]. There are a variety of diagnostic methods are available for DSPN [11,12]. Some of the popular clinical diagnostic methods for DSPN include vibration sensation test using a 128 Hz tuning fork [11], monofilament test [11], quantitative sensory testing (QST) [14], skin biopsy [15], nerve conduction studies (NCS) [16], corneal confocal microscopy (CCM) [17], electromyography (EMG) [18], etc. Several verified composite scores are available for the identification and stratification of DSPN severity [19]. The composite scoring systems are used to diagnose the different levels of DSPN among DM patients regularly as they are mostly based on tests on signs and symptoms using simple clinical tests. The most commonly used composite soring methods in both epidemiologic studies and clinical research: Michigan Neuropathy Screening Instrument (MNSI), Diabetic Neuropathy Symptom Score (DNS), Neuropathy Deficit Score (NDS) of Boulton, Toronto Clinical neuropathy Score (TCNS), and Neuropathy Impairment Score (NIS) [20]. In the Toronto consensus 2009, the use of composite neuropathy score is recommended for DSPN severity identification [9]. Even though a variety of composite scoring methods and clinical tests are available for identification and stratification of DSPN, there is still lacking uniformity and agreement in clinical research for DSPN severity classification [11] and is highly dependent on specialized personals. Moreover, due to the lack of standard methods, different countries use different composite scores. Therefore, a validated and standard screening tool is required for DSPN severity classification which can be used globally.

Recently, due to the enormous development in machine learning (ML) methods, their application in solving different disease classification problems are expanding [21,22,23,24]. Alike other diseases, development of an intelligent diagnosis system for DSPN have caught the interests of researchers because of the long term and severe complication arises due to DSPN. Currently, much attention is being received by CCM, which is a relatively new, non-invasive, and regenerative technique for DSPN diagnosis by using images of human corneal in-vivo. Much research is being conducted, emphasizing the automation of the CCM system using ML for a more accurate, reliable, and regenerable diagnosis of DSPN [25,26,27]. However, as CCM uses corneal images for identifying DSPN, it requires specialized personal and equipment which made it difficult to be available in regular healthcare facilities. In the initial stage, composite scores (NDS, MNSI, etc.) are widely used for screening DSPN signs and symptoms [12]. Intelligent systems using these DSPN scoring techniques can be a potential solution to solve the uniformity agreement problem with the DSPN severity grading due to their ability of reliable, accurate, reproducible diagnosis. In literature, few intelligent systems—such as fuzzy inference system (FIS) [28,29,30], multicategory support vector machine (SVM) [31], and adaptive fuzzy inference system (ANFIS) [32]—are being reported to use composite scoring methods for stratification of DSPN severity. The studies reported DSPN classifiers using fuzzy systems are not reliable because the FIS works relaying on the if–then rule base set by the experts or research based on human experience, Kazemi et al. [31], developed a multiclass SVM based DSPN severity classifier using NDS; however, their reported accuracy was only 76%. Fahmida et al. [32] have developed an ANFIS system for DSPN severity classification using MNSI with an accuracy of 91%, however, they have only considered three MNSI variables (questionnaire, vibration perception, and tactical sensitivity) to design their model. MNSI has been recommended on the position statement by ADA for the clinical diagnosis of DSPN [11]. The MNSI is very simple, inexpensive, and can be managed by any healthcare professional treating diabetic patients. The reliability and accuracy of the MNSI have been discussed elsewhere [10,33]. Therefore, this research proposes an ML-based DSPN severity classifier using MNSI.

In literature, conventional ML algorithms such as support vector machines (SVM) [34], k-nearest neighbor (KNN) [35], random forest (RF) [36], and artificial neural network (ANN) [37] are being used in different diseases diagnosis problems. Although the application of ML in disease diagnosis is a very common and well-established field of research, the application of ML in DSPN diagnosis using composite scoring techniques like MNSI is very limited in the existing literature. More studies are required to understand the performance of different ML techniques in DSPN diagnosis and stratification. In this research, we aim to observe the performance of eight different conventional ML algorithms: support vector machine (SVM), k-nearest neighbor (KNN), random forest (RF), discriminant analysis classifier (DAC), ensemble classifier (EA), naive Bayes (NB), linear regression (LR), and artificial neural network (ANN) for severity classification of DSPN using MNSI. A descriptive statistical analysis will be performed to evaluate the performance of these algorithms in DSPN severity classification. We aim to classify the DSPN patients into four severity classes: absent, mild, moderate, and severe classes with a good classification accuracy using different conventional ML.

The novelty of this research work is the implementation and performance analysis of different conventional ML-based intelligent classifiers that will be able to classify DSPN severity levels using MNSI scores. This study will benefit DSPN patients as well as diabetic patients with accurate, reliable, and early identification and stratification of DSPN and will help to received early treatments to prevent severe complications like ulceration and amputation. As the classifiers will be using the MNSI, it can be used with regular checkups in normal healthcare centers. This study will also investigate the effect of MNSI variables on DSPN severity classification using feature ranking. This study will investigate the best performing ML algorithms with different MNSI variable combinations in the stratification of DSPN. Still now, the identification and stratification of DSPN are based on offline analysis by the experts. This study can support health professionals in accurate, reliable, and real-time decision-making. Also, the problems due to lack of uniformity and agreements in the severity grading by different experts can be solved using an ML-based intelligent DSPN severity classifier. Therefore, this research aims to analyze the performance of different conventional ML-based intelligent classifiers for screening and stratification of DSPN severity and find the best performing classifier and MNSI variables.

## 2. Materials and Methods

### 2.1. Data Acquisition

In this research data was collected from the Epidemiology of Diabetes Interventions and Complications (EDIC) clinical trials which are conducted by the National Institute of Diabetes, Digestive and Kidney Diseases (Bethesda, Maryland, USA) to annually assess DSPN among type 1 diabetic patients since 1994 [38,39]. This clinical trial is still under continuous process which initially started with 1375 patients. Eight EDIC years of MNSI data were collected with 10,543 samples in total. MNSI is used to annually screen DSPN among the enrolled participants in EDIC trials.

### 2.2. Data Imputation

The MNSI dataset had a total of 10,543 samples, with 363 blank entries. After removing the blank entries, 10,180 samples were retrieved after removing the blank entries with missing values for MNSI variables. The k-nearest neighbor [35] data imputation technique had been used to fill the missing data.

### 2.3. Data Augmentation

The imputed EDIC dataset with 10,180 samples was imbalanced. Duplicate data were removed from the dataset keeping the first combinations only. Synthetic Minority Oversampling Technique (SMOTE) technique [40] had been used to balance the dataset with no overfitted data. Python 3.7 in-house written code was used for data imputation and augmentation.

### 2.4. DSPN Severity Scoring for MNSI

There are two parts to the MNSI [10] scoring system. The first part is a questionnaire consists of 15 yes/no questions related to the patient’s symptoms. The second part consists of five clinical examinations: the appearance of the foot (AF), ulceration (Ulc), ankle reflection (AR), vibration perception (VP), and tactile sensitivity (TS) are included in the clinical tests. The detailed scoring mechanism is described in [10]. In this study, a total of six MNSI variables (Questionnaire, AF, Ulc, AR, VP, and TS) were used. The preprocessed MNSI dataset was graded using the scoring technique proposed by Watari et al. [30]. The scoring was ranged from 0 to 10 and the severity classes are divided as follows:

(i). x ≤ 2.5: absent neuropathy
(ii). 2.5 < x < 5.0: mild neuropathy
(iii). 5.0 ≤ x < 8.0: moderate neuropathy
(iv). x ≥ 8.0: severe neuropathy

### 2.5. Feature Ranking

The eXtreme Gradient Boosting (XGBoost) [41] algorithm-based feature ranking model was developed to observe the effects of MNSI variables for DSPN diagnosis. XGBoost is a decision-tree-based ensemble Machine Learning algorithm that is capable of finding the effectiveness of different features from a prediction model. The preprocessed dataset was used to rank the MNSI features according to their impact on DSPN identification. The design of the XGBoost model has been discussed in our previous study [32].

### 2.6. ML Model Development Using MNSI Data

This study focus on performance analysis of different conventional ML algorithm based DSPN severity classifier using MNSI variables. Here we have considered eight conventional and supervised ML algorithms: support vector machine (SVM), k-nearest neighbor (KNN), random forest (RF), discriminant analysis classifier (DAC), ensemble classifier (EA), naive Bayes (NB), linear regression (LR), and artificial neural network (ANN). All the ML models were designed using MATLAB ver. R2020a, (The MathWorks, Inc., Natick, Massachusetts, MA, USA) with two different input combinations from the MNSI variables and DSPN severity level as output. Stratified 10-fold cross-validation was used to train and test the designed ML models. The performance of the designed ML models was evaluated using confusion matrices and the calculation of different performance parameters. A multiclass SVM model had been considered in this work. KNN model was designed for 20 nearest neighbors. RF model was designed with a 100 bagged decision tree. ANN was designed with 100 hidden layers and trained for 100 epochs for each fold.

### 2.7. Statistical Analysis

For Statistical analysis SPSS software (version 21.0; SPSS Inc., Chicago, Illinois, IL, USA) was used. All the statistical analyses for baseline characteristics of the EDIC patients were performed based on the DSPN and Non-DSPN groups and expressed as mean ± standard deviation (SD). Analysis of variance (ANOVA) was used to find out the statistical significance of the variables. An independent *t*-test was used to find out the 95% confidence intervals (95% CI). Pearson’s correlation coefficient was used to find out the correlation between different variables with DSPN classes. For the performance analysis of the ML models, ANOVA was used to find the statistical significance, Cohen’s kappa statistic [42] was used to find the reliability of the performance of the ML models, and Matthews Correlation Coefficient [43] was used to find the correlations between the observed and predicted classifications. Statistical significance was considered at *p* < 0.05.

## 3. Results

### 3.1. MNSI Dataset

**Figure 1:**
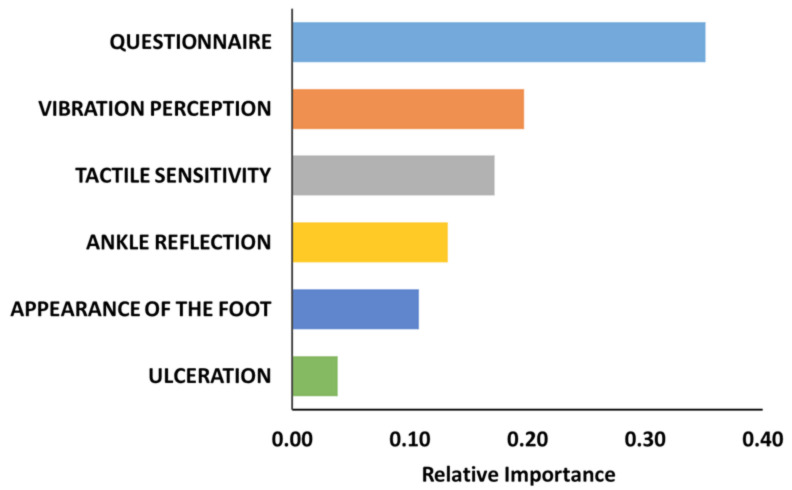
Relative importance of the MNSI variables using the XGBoost feature ranking technique [32].

EDIC patients’ baseline demographic variables have been observed to understand the characteristics of the patients and been shown in Table 1. The EDIC patients’ average age in the first year was 35.93 ± 6.945 years (657 male, 598 female), and the mean diabetic duration was 14.56 ± 4.906 years. Initially, we could have retrieved 957 non-neuropathic patients and 298 neuropathic patients, a total of 1255 patients’ data from the first year of the EDIC trials. 8 year of EIDC dataset there was 8819 absent, 1075 mild, 245 moderate, and 40 severe samples. After processing the EDIC dataset by data imputation and augmentation techniques, the final data set was prepared with 1200 samples per class. As per our previous study [32], we have observed the importance index of all MNSI variables using the XGBoost model. From Figure 1 we can observe that the questionnaire has an important index of 0.35 whereas clinical tests are ranked as VP, TS, AR, and AF based on the importance index in between 0.10 to 0.20 and Ulc has the lowest index of 0.5 [32].

Two datasets were prepared based on the feature ranking results. The first dataset (dataset-1) consists of the top three MNSI variables from feature ranking—i.e., questionnaire, VP, and TS—were considered as input variables to training the ML models. Also, one study by Watari et al. [30] used these three variables to classify DSPN patients’ severity using a fuzzy system. In the second dataset (dataset-2), all six MNSI variables were considered as inputs (questionnaire, AF, Ulc., AR, VP, TS) to train the ML models. Therefore, dataset-1 consisted of three inputs: VP and TS scores with a range from 0 to 2, questionnaire score with a range from 0 to 13, and one output: DSPN severity levels, 0: absent, 1: mild, 2: moderate, 3: severe neuropathic and dataset-2: consists of six variables: vibration perception, tactile sensitivity, ankle reflection, the appearance of feet and ulceration, ranging from 0 to 2 for each test and questionnaire with range 0 to 13, and one output: DSPN severity levels (0,1,2,3).

### 3.2. Performance Evaluation of ML Models

Two datasets were used for training eight conventional ML models—i.e., RF, SVM, EA, KNN, DAC, NB, LR, ANN, for DSPN severity classifiers—in total 16 models were trained. In the classification models, 10-fold stratified cross-validation was used and in case, 9-fold was used as train data, and 1-fold with 120 samples per class as test data. Table 2 and Table 3 are showing the performance evaluation of ML-based DSPN severity classifiers for 10-fold cross-validation using dataset-1 and dataset-2, respectively. Figures S1 and S2 (Supplementary Materials) are showing the confusion matrix for all the ML classifiers using dataset-1 (Table 2) and dataset-2 (Table 3). For dataset-1, RF has better accuracy (91.87 ± 1.42), sensitivity (91.8 ± 1.66), specificity (97.23 ± 0.55) compared with other ML algorithms, afterward, ANN, and SVM showed second-best performance for the dataset-1. All these three exhibit high correlation coefficients and substantial reliability based on kappa value. All the ML classifiers outputs showed a statistically significant relationship with test sets results.

For dataset-2, RF has better accuracy (98.50 ± 0.74), sensitivity (98.58 ± 1.67), specificity (99.50 ± 0.24) compare with other ML algorithms, afterward, SVM and EA showed second-best performance for the dataset-2. However, ANN showed poor performance for dataset-2 with 10-fold cross-validation and having a high standard deviation in performance parameters. All these three ML (RF, SVM, and EA) exhibits high correlation coefficients and near-perfect reliability based on kappa values. All the ML classifiers outputs showed a statistically significant relationship with test sets results for dataset-2. From Table 2 and Table 3, it is visible that all the ML classifiers’ performance enhanced with dataset-2 in comparison with dataset-1.

## 4. Discussion

Diabetic peripheral neuropathy (DSPN) is one of the major length-dependent complications of diabetic mellitus (DM). Since the 1900s, researchers are going on to establish a standardized diagnosis method for DSPN. To date, diagnosis, and severity stratification of DSPN requires manual grading by specialized expertise which are always subjective and vary depending on different screening methods. According to the study [7], less than one-third of health physicians would be able to identify the signs of DSPN, resulting in misleading diagnoses, contributing to high rates of morbidity and mortality. Although a variety of screening and diagnosing methods are accessible for DSPN, most of them require expensive equipment and specialized personnel to analyze the results from these tests; some of the methods are invasive and painful; some are not reproducible, and some have contradictory outcomes due to lack of standardization in diagnosis measures. Moreover, among the health professionals, there is a lack of understanding regarding the thorough diagnosis, controlling, and treatment process for DSPN [8]. Therefore, for early identification and satisfaction of the DSPN severity, a simple, cost-effective, reproducible, accurate diagnosis method is necessary, which can be globally applicable to solve the lack of understanding among the health professionals regarding DSPN.

Nowadays machine learning approaches are being researched in different aspects of healthcare systems due to their advantage of flexibility, cost-effectiveness, self-learning capacity, and being able to work as a second helping system for health professionals with accurate and reliable performance. Intelligent healthcare systems are capable of providing better patient satisfaction, helps health professionals with accurate, reliable, and real-time diagnosis, thus improving the healthcare facilities for DM patients. Intelligent systems using ML algorithms have now been widely researched for different biomedical systems and special importance is given to its application for disease diagnosis and minimization of health risks [21,22,23,24,44,45,46,47,48]. Alike other life-threatening diseases, DSPN has also caught the researchers’ attention for the development of an artificial intelligence-based diagnosing system for DSPN [25,26,27,28,29,30,31,32,48].

In literature, detection and stratification of DSPN severity have been reported using the FIS, ANFIS, SVM, and ANN algorithms [28,29,30,31,32,48]. DSPN exhibits non-linear characteristics and progresses differently in every patient. As FIS is developed using the if–then rule base, there is a chance of having human error and reliance on expert knowledge in characterizing the non-linear DSPN characteristics, thus the performance can be biased. Duckstein et al. [28] used electrophysiological examination for diabetic neuropathy classification using a fuzzy inference system. Picon et al. [29] have used four input variables including symptom assessment, sign examination from MNSI and diabetic duration, and HbAc1. They proposed a fuzzy inference system that was based on expert knowledge. Watari et al. [30] also have used the same fuzzy model to classify DSPN into four classes and have considered only 3 MNSI parameters including system assessment, vibration perception, and tactile sensitivity as the model input. However, as fuzzy works with if–then rules, it requires professionals training to set the rules for the fuzzy system, which can vary as to its subjective to healthcare professionals evaluation thus have a chance of having human errors. Kazemi et al. [31] developed a multicategory-based SVM model for DSPN severity classification based on NDS; however, the performance of the model is not reliable and reported an accuracy of 76%. In the study [32], an ANFIS based DSPN severity classifier was designed using the same three MNSI variables that have been proposed in [30]. This study has reported an accuracy of 91% using the three MNSI variables, whereas, in our study, we have observed that, the results got improved significantly when all the MNSI variables were considered to design ML models. In a study [48], the ANN model was developed for the diagnosis of DSPN using NCS, but no severity classification had been studied.

This research aims to develop different conventional ML-based DSPN severity classifiers for accurate and reliable stratification of DSPN severity. Here eight conventional ML models—i.e., SVM, KNN, RF, EA, NB, DAC, ANN, and LR—were trained for the classification of DSPN patients into four severity groups: absent, mild, moderate, and severe. In this study, we only have considered the conventional machine learning models for developing the severity classifiers. Deep learning models have not been studies here as they are being widely used in complex classifications and regression problems where the data have high dimensions and complex features [49]. As we intend to develop a simple and cost-effective DSPN severity classifier, using deep models can introduce higher costs due to its complex computational models [50].

As DSPN exhibits non-linear characteristics, the data to train the model plays a crucial role. The performance of the ML models will depend on how well the data is showcasing the real situation. Therefore, for better accuracy of the models, we have considered a database from the EDIC trial, which is a large and continuous clinical trial, uses MNSI to follow-up the enrolled patients’ DSPN condition annually [10,38,39]. As models were trained with a real dataset, it can accurately learn the non-linear characteristics of DSPN. As the MNSI variables are semi quantitation or non-quantitative tests, it can be easily deployed in any regular healthcare facility. As the EDIC trials consist of a wide range of demographic variables from 29 different clinical centers, this dataset is realistic in observing different classes of DSPN severity with a variety of populations.

Two datasets were used to train the ML models. For both of the datasets, the RF model was working better in comparison with other ML models used in this study. For models training dataset-1—i.e., top three MNSI variables from feature ranking—the performance of the ML models can be ranked as RF > EA > ANN > SVM > KNN > NB > DAC > LR. All the ML models using dataset-1 showed substantial reliability with kappa values between 0.66 to 0.78 [42] states that, the inputs used in dataset-1 are moderately accurate to identify DSPN severity. From the performance analysis for different ML models, it can be seen that only three variables are not enough to accurately identifying DSPN severity even though these variables got high importance index based on feature ranking. From dataset-2, where we have considered all the MNSI variables exhibit that, all the ML models exhibited very good performance except ANN. ANN performance has not been improved much after using all the MNSI variables and has a higher standard deviation in performance, indicating that, in some of the folds from the cross-validation process where ANN was not able to train properly and had poor performance. For dataset-2, ML models performance can be ranked as RF > SVM > EA > KNN > NB > DAC > LR > ANN. Also using all six MNSI variables to train ML models, the kappa values for the models were between 0.89 to 0.98 which indicates that the models are in perfect agreement [42] with the data and the variables used in dataset-2 are perfectly accurate to identify DSPN using ML models. Predicted classes by ML models and the true classes using dataset-2 have a higher correlation in comparison with dataset-1. From this study, we can recommend that all the six MNSI variables need to be considered while DSPN severity grading for higher accuracy of the model’s performance.

According to the International Diabetic Federation [1] in 2019 almost 463 million people are affected with diabetes and 50% of the total prevalence is suffering from DSPN. USD 760 million is spent on diabetics and the health expenditure for diabetic patients increases with severity [1,51]. By enhancing the awareness among patients about DSPN as well as the performance of the diagnosis methods will help to improve the healthcare facilities for diabetic patients. As almost 50% of the DM patients are affected by DSPN at some point of DM duration, the global expenditure can be significantly reduced if an improved, cost-effective, accurate, reliable diagnosis method can be deployed which will be able to help with real-time DSPN severity identification and will allow early detection and treatment of diabetic neuropathy as well as prevent from severe complications like foot ulceration and amputation. ML algorithms based on DSPN severity classifiers are capable of providing all these benefits to DM patients. It will also be beneficial in overcoming the shortcomings in the available conventional diagnosis methods which relied on offline analysis by healthcare professionals, leading to a delayed and sometimes biased diagnosis for DSPN. The analysis results showed that RL models outperforming the other ML models with all MNSI variables for DSPN severity classification. This RF based DSPN severity classifier can be used as a support system for the healthcare professionals in more accurate, reliable, and faster identification and stratification of DSPN. A limitation of this study is that it had been conducted using the EDIC dataset, which only recruited type-1 diabetic patients. The effect of DSPN in type-2 patients and their severity classification using MNSI still need to be studied. Nerve conduction studies (NCS) have been considered the gold standard for DSPN identification and stratification. In the future, we aim to use NCS and other risk factors for DSPN with MNSI for severity identification and stratification using ML models. In the future, a prediction system can be incorporated with an RF-based DSPN classifier so that health professionals will be able to predict patients’ future conditions using patients’ previous and present conditions. This will help to identify the high-risk individuals ahead of time so that proper treatment can be provided to the patients to avoid extreme situations.

## 5. Conclusions

DSPN is one of the most common forms of diabetic neuropathy (DN) and almost 90% of the DN patients suffer from it. Diagnosis of DSPN is complicated because of contradictory and subjective diagnosis techniques.

Although many diagnoses and composite scoring techniques have been reported and many studies are being conducted to validate these systems, yet it lacks consistency and is sensitive to population size. To overcome this issue, machine learning techniques can be a good solution. The application of ML in different aspects of the biomedical sector has shown a promising impact in improving the performance from the usual methods. In this research, we have studied the performance of different conventional ML techniques (RF, SVM, KNN, EA, NB, DAC, ANN, LR) in the diagnosis and stratification of DSPN. We have using the MNSI composite scoring technique for DSPN diagnosis and observed the importance of the MNSI variables on DSPN identification and stratification. From this analysis, we have found that the random forest algorithm with all MNSI variable model works better in DSPN stratification. Therefore, a random forest based MNSI scoring technique can help health care professionals to identify DSPN patients and grade their severity. This type of system can overcome the problem of inconsistency and lack of agreement between professionals with diagnostic criteria for DSPN.

## Figures and Tables

**Table 1:** Baseline characteristics of the EDIC patients.

*N* = 1255	Mean	Std. Error Mean	Min	Max	95% Confidence Interval		r	*p*	f
					Lower Limit	Upper Limit			
Age (years)	35.95 ± 6.93	0.20	20.42	50.99	35.57	36.34	0.18	<0.05	43.96
Diabetic duration (years)	14.51 ± 4.92	0.14	7.08	26.92	14.24	14.78	0.12	<0.05	18.40
Hba1c (%)	8.22 ± 1.39	0.04	0.00	14.00	8.14	8.30	0.10	0.95	12.54
HDL Cholesterol (mg/dL)	52.559 ± 15.98	0.45	0.00	103.0	51.67	53.44	0.002	<0.05	0.01
LDL Cholesterol (mg/dL)	110.68 ± 36.48	1.03	0.00	235.0	108.7	112.7	0.07	0.21	5.62
BMI (kg/m^2^)	26.17 ± 4.05	0.11	16.63	39.48	25.94	26.39	0.04	<0.05	1.56
Hypertension (%)	0.23 ± 0.42	0.01	1.00	0.16	0.20	0.25	0.16	<0.05	31.75

**Table 2:** Performance analysis of different ML classifiers for DSPN severity classification using Dataset-1.

	Accuracy (%)	Sensitivity (%)	Specificity (%)	Precision (%)	F1-Score (%)	False Positive Rate	Fold Error (%)	Matthews Correlation Coefficient	Kappa	*p* *
RF	91.87 ± 1.42	91.8 ± 1.66	97.23 ± 0.55	91.98 ± 1.54	91.8 ± 1.65	2.75 ± 0.55	8.25 ± 1.66	0.89	0.78	<0.001
ANN	90.00 ± 1.53	89.76 ± 1.21	96.59 ± 0.41	89.76 ± 1.19	89.67 ± 1.21	3.43 ± 0.41	10.26 ± 1.21	0.9	0.7	<0.001
SVM	89.69 ± 1.75	89.72 ± 1.32	96.56 ± 0.44	89.96 ± 1.21	89.74 ± 1.3	3.45 ± 0.44	10. 37 ± 1.32	0.9	0.7	<0.001
EA	89.98 ± 1.83	90.02 ± 1.45	96.67 ± 0.48	90.0471 ± 1.47	89.95 ± 1.45	3.33 ± 0.48	9.99 ± 1.45	0.87	0.73	<0.001
KNN	87.81 ± 2.14	88.07 ± 0.86	96.03 ± 0.28	88.42 ± 0.92	87.85 ± 0.88	3.99 ± 0.28	11.95 ± 0.87	0.84	0.68	<0.001
DAC	87.21 ± 1.53	87.22 ± 1.31	95.75 ± 0.44	87.24 ± 1.36	87.06 ± 1.34	4.25 ± 0.44	12.8 ± 1.31	0.83	0.66	<0.001
NB	87.81 ± 1.17	87.82 ± 1.17	95.95 ± 0.39	87.94 ± 1.23	87.671 ± 1.18	4.07 ± 0.39	12.2 ± 1.17	0.84	0.68	<0.001
LR	87.21 ± 1.17	94.17 ± 2.32	94.47 ± 0.73	87.81 ± 1.17	87.87 ± 1.20	4.06 ± 0.39	0.12 ± 0.01	0.84	0.68	<0.002

^*^p values were calculated using ANOVA tests in between the actual and predicted outcome and the cut-off was set as *p* < 0.05.

**Table 3:** Performance analysis of different ML classifiers for DSPN severity classification using Dataset-2.

	Accuracy (%)	Sensitivity (%)	Specificity (%)	Precision (%)	F1-Score (%)	False Positive Rate	Fold Error (%)	Matthews Correlation Coefficient	Kappa	*p* *
RF	98.50 ± 0.74	98.58 ± 1.67	99.50 ± 0.24	98.52 ± 0.73	98.50 ± 0.74	0.50 ± 0.24	1.50 ± 0.74	0.98	0.98	<0.001
SVM	97.38 ± 0.91	98.33 ± 0.56	99.13 ± 0.31	97.40 ± 0.91	97.37 ± 0.91	0.87 ± 0.31	2.63 ± 0.91	0.97	0.97	<0.001
EA	96.04 ± 0.79	97.58 ± 1.33	98.68 ± 0.26	96.09 ± 0.75	96.04 ± 0.79	1.32 ± 0.26	3.96 ± 0.79	0.96	0.95	<0.001
KNN	95.46 ± 0.93	94.58 ± 1.97	98.49 ± 0.31	95.48 ± 0.94	95.42 ± 0.95	1.52 ± 0.31	4.54 ± 0.93	0.95	0.94	<0.001
DAC	93.23 ± 1.39	95.42 ± 1.06	97.74 ± 0.46	93.42 ± 1.33	93.21 ± 1.40	2.26 ± 0.46	6.77 ± 1.39	0.93	0.91	<0.001
NB	91.67 ± 1.56	97.00 ± 1.93	97.22 ± 0.52	91.76 ± 1.49	91.64 ± 1.55	2.78 ± 0.52	8.33 ± 1.56	0.92	0.89	<0.001
LR	91.66 ± 1.51	95.50 ± 1.04	99.19 ± 0.65	91.67 ± 1.56	91.71 ± 1.52	2.78 ± 0.52	8.23 ± 1.67	0.89	0.78	<0.001
ANN	90.06 ± 13.0	90.06 ± 13.0	96.69 ± 4.34	90.79 ± 11.0	88.86 ± 16.6	3.31 ± 4.34	9.94 ± 13.02	0.89	0.87	<0.001

^*^p-values were calculated using ANOVA tests in between the actual and predicted outcome and the cut-off was set as *p* < 0.05.

## Data Availability

Restrictions apply to the availability of these data. Data was obtained from National Institute of Diabetes and Digestive and Kidney Diseases (NIDDK) (Bethesda, MD, USA) and are available (https://repository.niddk.nih.gov/studies/edic/ accessed on 28 April 2021) with the permission of NIDDK.

## Supplementary Materials

The following are available online at https://www.mdpi.com/article/10.3390/diagnostics11050801/s1, Figure S1. Confusion matrices for (a) RF, (b) SVM, (c) EA, (d) KNN, (e) DAC, (f) NB, and (g) ANN models for classification of DSPN severity classes using dataset-1 (0: absent or non-neuropathic, 1: mild neuropathic, 2: moderate neuropathic, 3: severe neuropathic), Figure S2. Confusion matrices for (a) RF, (b) SVM, (c) EA, (d) KNN, (e) DAC, (f) NB, (g) ANN models for classification of DSPN severity classes using dataset-2 (0: absent or non-neuropathic, 1: mild neuropathic, 2: moderate neuropathic, 3: severe neuropathic).
