# Supplementary material for: Performance Analysis of Conventional Machine Learning Algorithms for Diabetic Sensorimotor Polyneuropathy Severity Classification
Source: Diagnostics (Basel). 2021 Apr 28;11(5):801. doi: 10.3390/diagnostics11050801 (PMC8146253; doi:10.3390/diagnostics11050801)
Supplement: Supplementary file 1 [file diagnostics-11-00801-s001.zip › diagnostics-1153709-supplementary.pdf]

Supplementary Materials

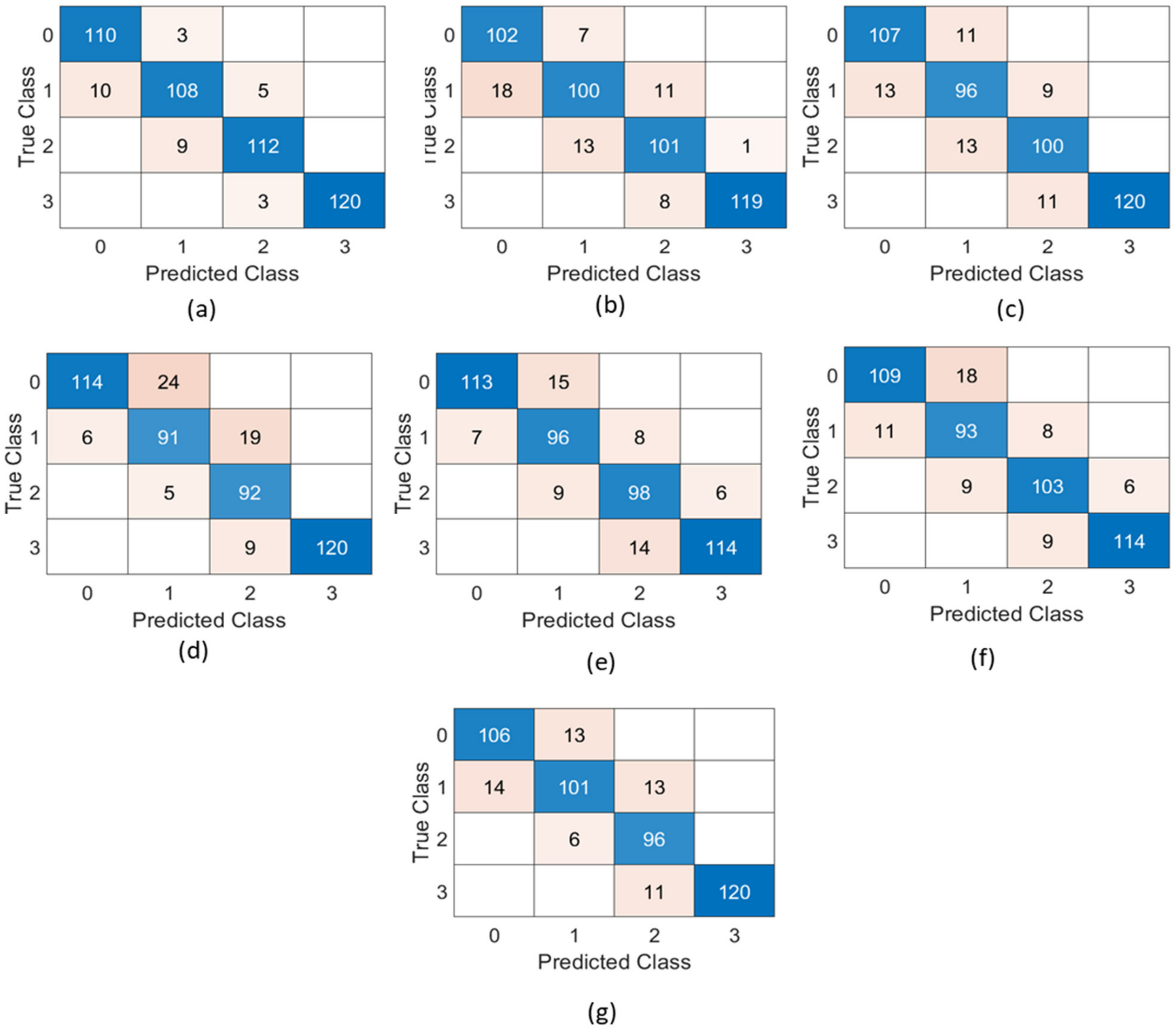

Supplementary Figure S1. Confusion matrices for (**a**) RF, (**b**) SVM, (**c**) EA, (**d**) KNN, (**e**) DAC, (**f**) NB, and (**g**) ANN models for classification of DSPN severity classes using dataset-1 (0: absent or non-neuropathic, 1: mild neuropathic, 2: moderate neuropathic, 3: severe neuropathic).

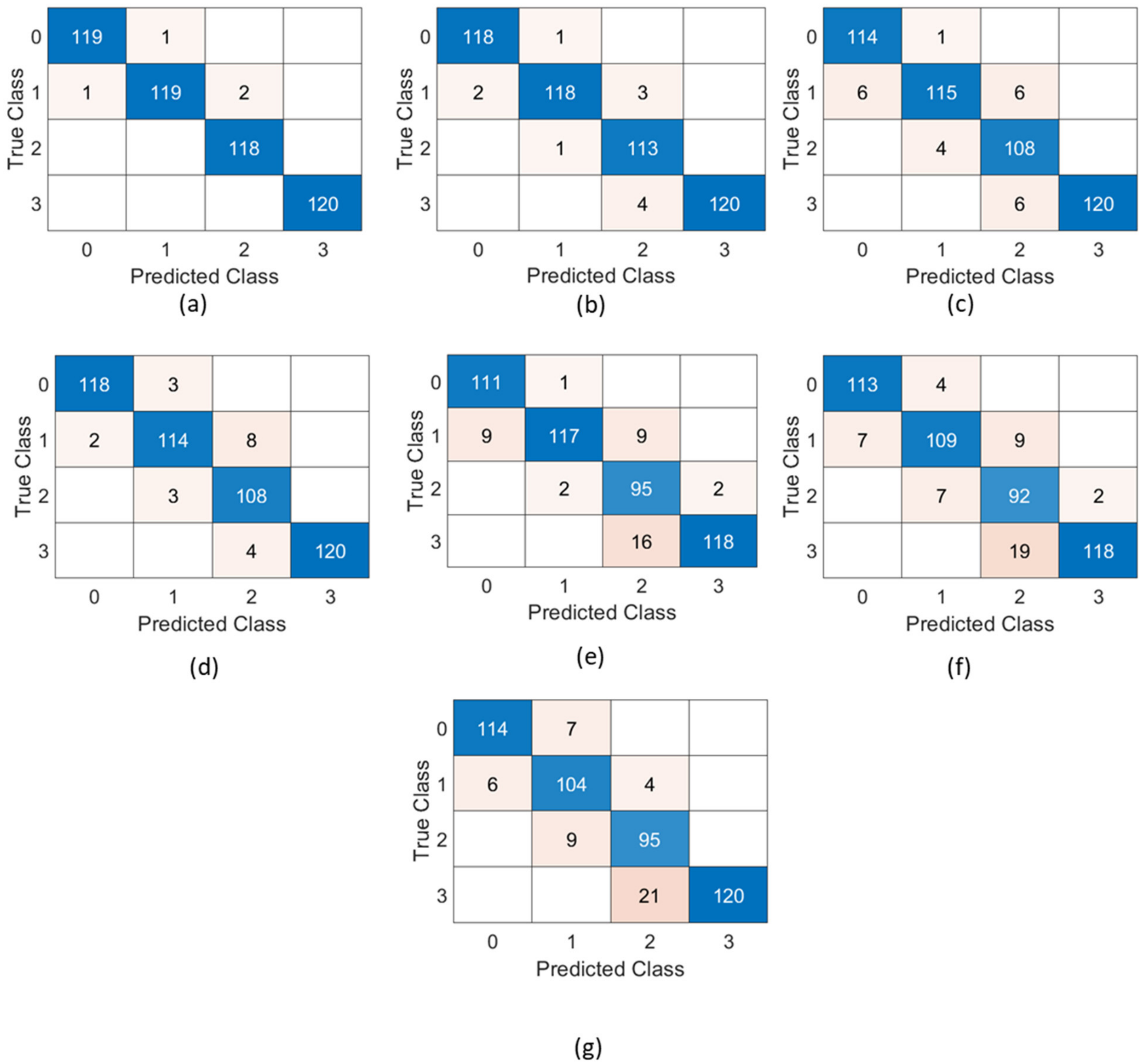

Supplementary Figure S2. Confusion matrices for (**a**) RF, (**b**) SVM, (**c**) EA, (**d**) KNN, (**e**) DAC, (**f**) NB, (**g**) ANN models for classification of DSPN severity classes using dataset-2 (0: absent or non-neuropathic, 1: mild neuropathic, 2: moderate neuropathic, 3: severe neuropathic).
